# Intramuscular fat in gluteus maximus for different levels of physical activity

**DOI:** 10.1038/s41598-021-00790-w

**Published:** 2021-11-01

**Authors:** Martin A. Belzunce, Johann Henckel, Anna Di Laura, Alister Hart

**Affiliations:** 1 Royal National Orthopaedic Hospital, Stanmore, HA7 4LP UK; 2 Institute of Orthopaedics and Musculoskeletal Science, Royal National Orthopaedic Hospital (RNOH), University College London, Brockley Hill, Stanmore, Middlesex, HA7 4LP UK

**Keywords:** Anatomy, Biomarkers, Medical imaging

## Abstract

We aimed to determine if gluteus maximus (GMAX) fat infiltration is associated with different levels of physical activity. Identifying and quantifying differences in the intramuscular fat content of GMAX in subjects with different levels of physical activity can provide a new tool to evaluate hip muscles health. This was a cross-sectional study involving seventy subjects that underwent Dixon MRI of the pelvis. The individuals were divided into four groups by levels of physical activity, from low to high: inactive patients due to hip pain; and low, medium and high physical activity groups of healthy subjects (HS) based on hours of exercise per week. We estimated the GMAX intramuscular fat content for each subject using automated measurements of fat fraction (FF) from Dixon images. The GMAX volume and lean volume were also measured and normalized by lean body mass. The effects of body mass index (BMI) and age were included in the statistical analysis. The patient group had a significantly higher FF than the three groups of HS (median values of 26.2%, 17.8%, 16.7% and 13.7% respectively, *p* < 0.001). The normalized lean volume was significantly larger in the high activity group compared to all the other groups (*p* < 0.001, *p* = 0.002 and *p* = 0.02). Employing a hierarchical linear regression analysis, we found that hip pain, low physical activity, female gender and high BMI were statistically significant predictors of increased GMAX fat infiltration.

## Introduction

An increase of intramuscular fat (IMF) in the muscles is associated with loss of strength and mobility, making it an important marker for muscle health^1–4^. A high content of IMF has been linked mainly to aging/sarcopenia^3–8^, but also to muscular^9,10^ and physiological pathologies^11–15^ and low levels of physical activity^16^.

IMF accumulation has been mainly evaluated in the thigh^2,4,5,7,10,16–21^ and calf muscles^3,16,22^, while the hip muscles have been overlooked despite their fundamental role in running, walking, standing and other human daily activities^23,24^. In the small number of studies looking at this muscle group, fatty infiltration in the glutei was found in patients with diseased hips^25,26^ and was also correlated with accidental falls in elderly people^19,27^. Of the gluteal muscles, gluteus maximus (GMAX) is particularly involved in physically demanding activities, such as in running, climbing or lifting^24,28,29^. However, to the best of our knowledge, GMAX fat content has not been studied for different levels of activity, including demanding physical tasks such as endurance running.

When compared to the reduction of muscle volume^2,8^, muscle composition changes, in particular an increase of IMF and intermuscular fat (IMAT), proved to be a better predictor for the loss of mobility and muscle strength observed with aging^2,7,16,22^. In addition, fat infiltration in the thigh and calves has been correlated with a reduction of physical activity in healthy young adults and obese adults^16^ with diabetes^30^. Identifying and quantifying differences in the IMF content of gluteus maximus (GMAX) in subjects with different levels of physical activity can provide a new tool to evaluate hip muscles health.

In this study, we aimed to better understand fat infiltration of GMAX. Our primary objective was to determine the association between IMF of GMAX and level of physical activity using a group of patients with hip pain, and three groups of healthy subjects with low, medium and high levels of physical activity. Our secondary objective was to determine the effect of female gender, older age, high BMI and hip pain/symptoms on the relationship between physical activity and fat infiltration of GMAX.

## Methods

### Study design

This was a cross-sectional study involving 70 subjects who underwent Dixon MRI of the pelvis. The study participants were recruited for a study looking at the effects of marathon running in the hip and were divided into three groups of healthy subjects (HS) and a group of patients. The inclusion criteria for the HS was absence of hip injury or hip surgery, and no contraindication to MRI. In the HS (n = 51), eight were no runners (doing no more than 90 min of physical activity per week), 27 were in the first weeks of a training programme for their first marathon (running recreationally at least 2 times a week at the time of the scan) and 16 were experienced runners that have run at least three marathons or ultra-marathons in the past.

The HS filled out a structured questionnaire regarding their training, where the number of hours per week of running and of other physical activities per week (cycling, swimming or resistance training) were registered. Using these data, the healthy subjects were regrouped into three groups: a) low activity: subjects doing less than 4 h per week of physical activity (Low); b) medium activity: subjects doing between 4 and 8 h per week (Mid); and c) high activity: subjects doing more than 8 h per week (High).

The patients’ group (n = 19) consisted of subjects with very low levels of physical activity due to hip pain. All of them had an MRI investigation requested after being assessed for hip pain by an orthopaedic surgeon. The MRI reports indicated a variety of injuries and hip conditions, being gluteus medius or hamstring tendinopathy (n = 7) and arthritis (n = 5) the most frequent findings. In only one case, fatty atrophy of the gluteal muscles was reported. All the patients completed the Oxford Hip Score (OHS) questionnaire^31^ and their hip dysfunction was graded as Severe (scores 0–19), Moderate (20–29), Mild (30–39) and Satisfactory (40–48) as recommended in the OHS system. We used these grades as a measure of the degree of hip pain. This group represented the subjects with lowest physical activity in our study. A detailed description of the patients group can be found in the Supplementary Table S1.

In terms of sample size, we aimed for approximately 16 subjects per group that is the sample size needed for a statistical power of 0.8 and an effect size of 3% in fat fraction (FF) between groups. To estimate this, we assumed that the standard deviation for each group was 4% (based on the preliminary results obtained for two heterogeneous groups in^32^).

The demographic characteristics of the four groups are shown in Table 1. All subjects provided written informed consent and the study was approved by the UCL Research Ethics Committee [13823/001]. All experiments were performed in accordance with relevant guidelines and regulations.

**Table 1:** Characteristics of the healthy subjects and patients groups.

	Patients	Healthy subjects		
	Hip pain (n = 19)	Low activity (n = 13)	Medium activity (n = 18)	High activity (n = 20)
Gender	M = 7, F = 12	M = 8, F = 5	M = 7, F = 11	M = 16, F = 4
Age (years)	49.9 (19–63)	31.8 (22–45)	35.2 (20–59)	31.4 (18–45)
Weight (kg)	77.0 (20.7)	67.9 (9.8)	69.0 (10.0)	73.8 (8.7)
Height (m)	1.67 (0.09)	1.75 (0.11)	1.72 (0.12)	1.76 (0.08)
BMI (kg/m^2^)	27.3 (6.0)	22.0 (1.9)	23.3 (3.4)	23.7 (2.0)

The age values correspond to mean (min–max) values, while mean (standard deviation) values are reported for the other variables.

### MRI acquisition

All subjects underwent a standardized MRI protocol. The MR images were acquired on a 3 T scanner (Siemens Magneton Vida, Erlangen, Germany) using a body coil. The scanning protocol consisted of standard clinical sequences for the hips and axial Dixon (slice thickness 1.5 mm, spacing between slices 1.95 mm, repetition time (TR) 4570 ms, echo time (TE) 45 ms, number of excitations 1, number of echoes 14, flip angle 120°) of the pelvis. The field of view (FOV) of the Dixon sequence covered axially from 1 cm below the lesser trochanter to the top of the iliac crest. The voxel size was 0.47 × 0.47 × 1.95 mm3. We used Dixon MRI because it provides a FF signal, obtained from fat and water images, which can be used to measure quantitatively IMF^33^. The Dixon FF signal has been validated and widely applied to quantify fat infiltration in organs and muscles^11,34,35^.

### Fat infiltration measurements

We estimated the GMAX IMF content using an automated tool that computes the FF of GMAX from Dixon images^32,36^. The tool is an in-house plugin for Simpleware ScanIP (Version 2020.6; Synopsys, Inc., Mountain View, USA) based on a multi-atlas segmentation method that employs a library with 15 manually segmented Dixon scans. Different to most of the studies looking at fat-content, where cross-sectional areas (CSA)^37–39^ of the muscles were used, our tool obtains the mean FF of the full 3D muscle volume. The method automatically generates 3D labels for left and right GMAX in the in-phase Dixon image, being a label the sets of voxels that represent each muscle in the image. Next, the mean GMAX FF value is computed by averaging the label voxel values of the FF image, being the latter the ratio image between the fat image and the sum of the water and fat images. In average, 1.2 × 10^6^ voxels were averaged per GMAX label. In^32^, we showed that the FF error for the comparison of two small groups of subjects was lower than 0.6%. The resulting mean FF value of each label quantifies GMAX IMF. The latter, which is the adipose tissue depot between and among skeletal muscle fibres in the skeletal muscle bed^18,40^, needs to be distinguished from IMAT, which is fat underneath the deep fascia and between adjacent muscle groups.

In order to standardize the measurement and to avoid regions with tendinous tissue, we computed the FF in the muscle bulk, which we defined as the region of the gluteus maximus that goes from the axial slice where the tip of the lesser trochanter (LT) is found to the slice at the level of the anterior superior iliac spine (ASIS). This muscle bulk definition was previously proposed in^36^. In addition, we measured GMAX volume and lean volume for the muscle bulk regions. The lean volume was defined as the volume multiplied by (1-FF). Both volume measurements were normalized by the lean body mass (LBM) of each subject using the Boer formula^41^.

**Figure 1:**
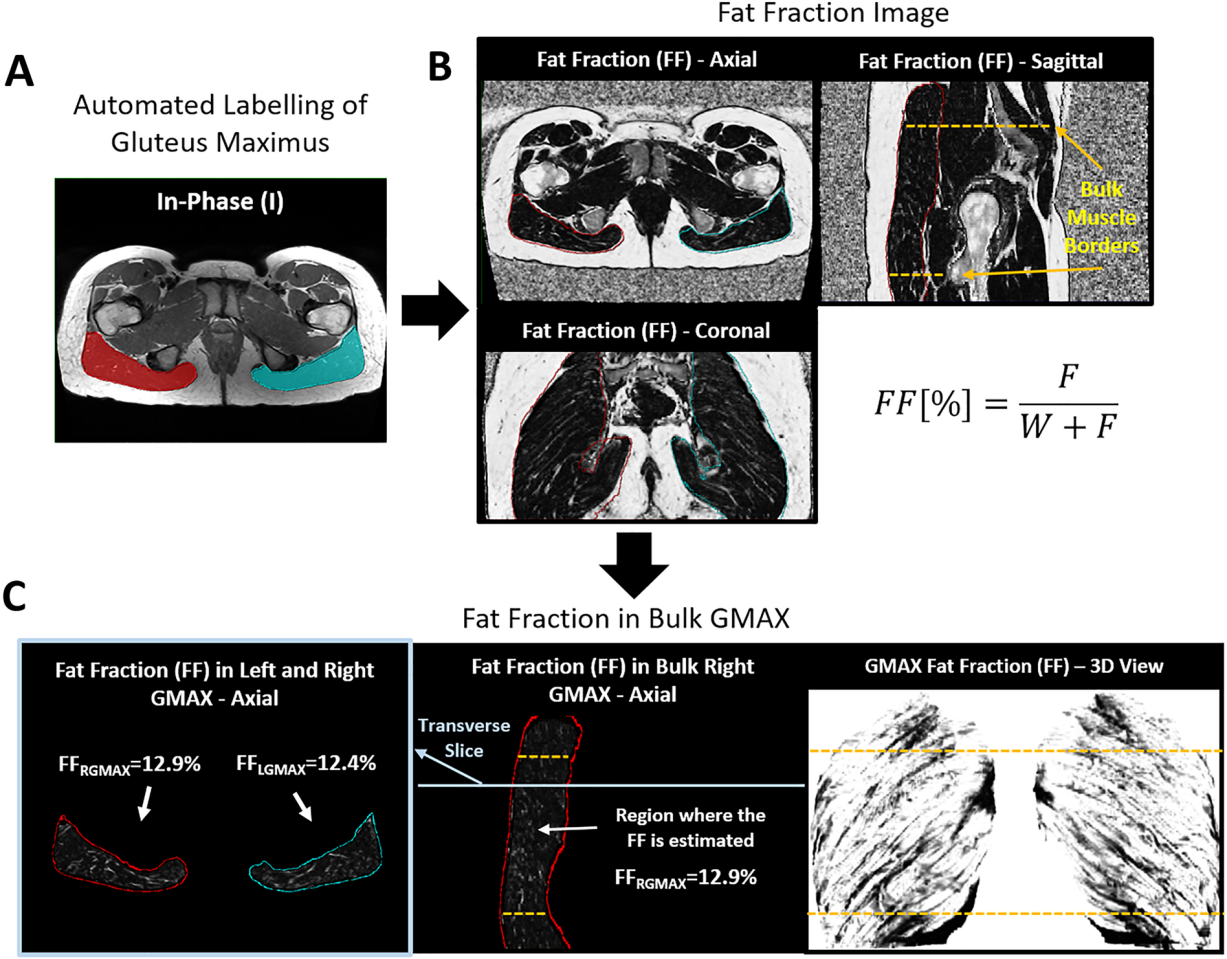
FF measurement of GMAX. (**A**) An automated tool is used to label the in-phase Dixon image. (**B**) A FF image is created from fat and water images where the labels are overlaid and landmarks for the bulk muscle are identified. (**C**) FF of right and left GMAX are estimated by masking the FF image with the labels. A coronal view of the masked FF image is shown, where the central region between dashed lines is used to estimate the FF. On the left an axial slice of the masked image (highlighted in the coronal view) is found. On the right, a 3D view of the FF in both gluteus maximus is shown where a dark signal indicates fat content.

Figure 1 shows a graphical example of the estimation of the FF in both the left and right GMAX from a Dixon scan. The automated labels for this example can be seen in A as translucent masks overlaid in an axial slice, in B the outline of each label is shown in axial, coronal and sagittal views. In the sagittal view, the slices that delimit the bulk muscle are shown with dashed lines. Slices and 3D views of the FF signal inside GMAX are shown in C.

### Statistical analyses

The FF values of GMAX were tested for normality within each group using a Shapiro–Wilk test. For some of the groups, the null hypothesis that the group’s samples followed a normal distribution was rejected. For this reason, we reported parametric [mean ± standard deviation (SD)] and non-parametric [median and interquartile range (IQR)] descriptive statistics for the FF values in each group, with and without considering gender.

The FF, volume and lean volume population distributions were first compared for the four groups with different levels of activity (Hip Pain, Low, Mid, High) using a Kruskal–Wallis test for not normally distributed samples. The mean value of left and right GMAX for each variable was used for this analysis. A multiple comparison test was executed using Tukey’s Honestly Significant Difference procedure to obtain more information of the Kruskal–Wallis test.

We also compared left and right GMAX FF for each activity group using a Wilcoxon signed-rank test. For the patient group, we additionally used Wilcoxon signed-rank test to compare the symptomatic to the pain free side.

We performed a hierarchical linear regression analysis to study which variables explained a significant amount of the GMAX FF variance. In this analysis, we built a set of linear regression models adding sequentially gender, BMI, age, level of physical activity, the OHS grading, muscle side and pain side as predictor variables, while the FF was the dependent variable. The left and right FF values were considered separately. We used a categorical variable for the level of physical activity and a numerical for the OHS grading (1 to 4). For the pain side variable, we used a categorical variable (on/off) indicating if there was pain on a given side. The models were estimated using MATLAB (Version R2018A, The Mathworks, Inc., USA) and each variable was included sequentially in each new model in the same order as they were described previously (i.e. the first model would only include gender as predictor variable). A variable was considered a FF predictor if it was statistically significant and its inclusion increased the R^2^ of the linear regression.

A post hoc power analysis was computed for the hierarchical linear regression using the G*Power software^42^. In this analysis, the power of the four physical activity categories was estimated taking into account the seven predictor variables (gender, BMI, age and the four physical activities categories).

Finally, we compared FF and volume metrics as predictors of physical activity using a multivariate logistic regression analysis. For this test, we grouped the study subjects into Not Active (hip pain and low level of physical activity groups) and Active (mid and high levels of physical activity). Gender, BMI and age were also included as independent variables. We computed the odds ratios (OR) with their 95% confidence intervals (CI) for each variable. A similar analysis was performed to compare if FF and volume variables were predictors of hip pain. In this case, we compared the patients group against all the healthy subjects.

We used a level of statistical significance (α) of 0.05 for all the tests.

## Results

The patient group had a significantly higher GMAX FF than the three groups of healthy subjects and the high activity group had lower GMAX FF than the low activity. In the hierarchical linear regression analysis, female gender, high BMI and low level of physical activity were all predictors of higher FF values, and therefore of GMAX IMF content; but the degree of hip pain was not. The normalized volume of GMAX was larger for the high activity group than for the patient and low activity groups, but no differences were found between the patients and the low and medium levels of activity groups. GMAX IMF was a predictor of physical activity, while GMAX volume was not.

### Gluteus maximus intramuscular fat and physical activity

**Figure 2:**
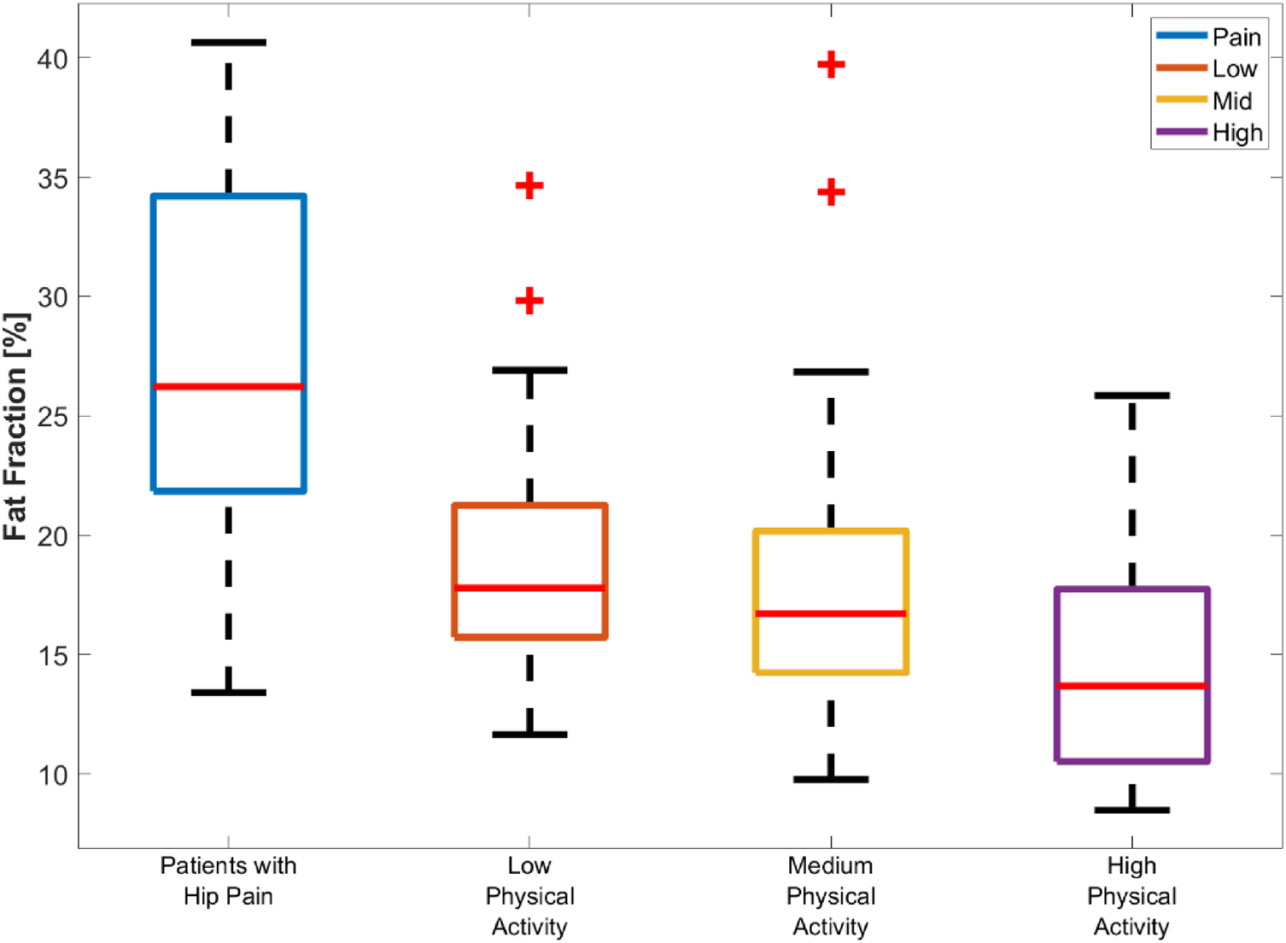
Boxplots of GMAX FF values for each group under study. On each box, the central mark is the median, the edges of the box are the 25th and 75th percentiles. The outliers are plotted individually. The patient group was significantly different to the three HS groups. Among the HS, the low and high activity groups were statistically different (*p* < 0.05).

The mean ± SD and median (IQR) FF values of GMAX are presented in Table 2 for the four different groups under study without and with gender differentiation. The mean FF values of right and left sides were used for each subject. Boxplots of FF for each of the groups are shown in Fig. 2. The non-parametric Kruskal–Wallis test showed statistically significant differences of GMAX FF between the different activity groups (*p* < 0.001). The post-hoc Tukey’s Honestly Significant Difference analysis indicated that the patient group was different to the three other groups (*p* = 0.002, *p* < 0.001 and *p* < 0.001 for Low, Mid and High respectively), while there was a significant difference only between the low and high activity groups (*p* = 0.03) for the healthy subjects when gender, BMI and age were not considered.

**Table 2:** Mean ± SD and Median (IQR) values for GMAX FF in the Patients, and Low, Mid and High activity groups and all healthy subjects together.

Group	Gender	Mean ± SD (%)	Median (IQR) (%)
Patients	Male	29.0 (7.8)	30.0 (22.9–34.8)
	Female	27.0 (7.7)	26.0 (21.5–32.9)
	All	27.7 (7.7)	26.2 (21.8–34.2)
Low physical activity	Male	16.9 (3.6)	16.4 (14.6–19.4)
	Female	22.6 (6.4)	20.4 (16.8–26.9)
	All	19.1 (5.5)	17.8 (15.7–21.3)
Medium physical activity	Male	14.1 (2.8)	14.6 (11.8–15.8)
	Female	20.4 (6.6)	19.5 (15.6–22.3)
	All	18.0 (6.3)	16.7 (14.2–20.2)
High physical activity	Male	14.0 (4.6)	13.1 (10.3–17.0))
	Female	17.3 (4.4)	17.5 (13.3–19.4)
	All	14.7 (4.7)	13.7 (10.5–17.7)
All healthy subjects	Male	14.8 (4.1)	14.6 (11.5–17.3)
	Female	20.4 (6.3)	18.9 (16.2–23.6)
	All	17.0 (5.8)	16.2 (13.0–19.6)

**Figure 3:**
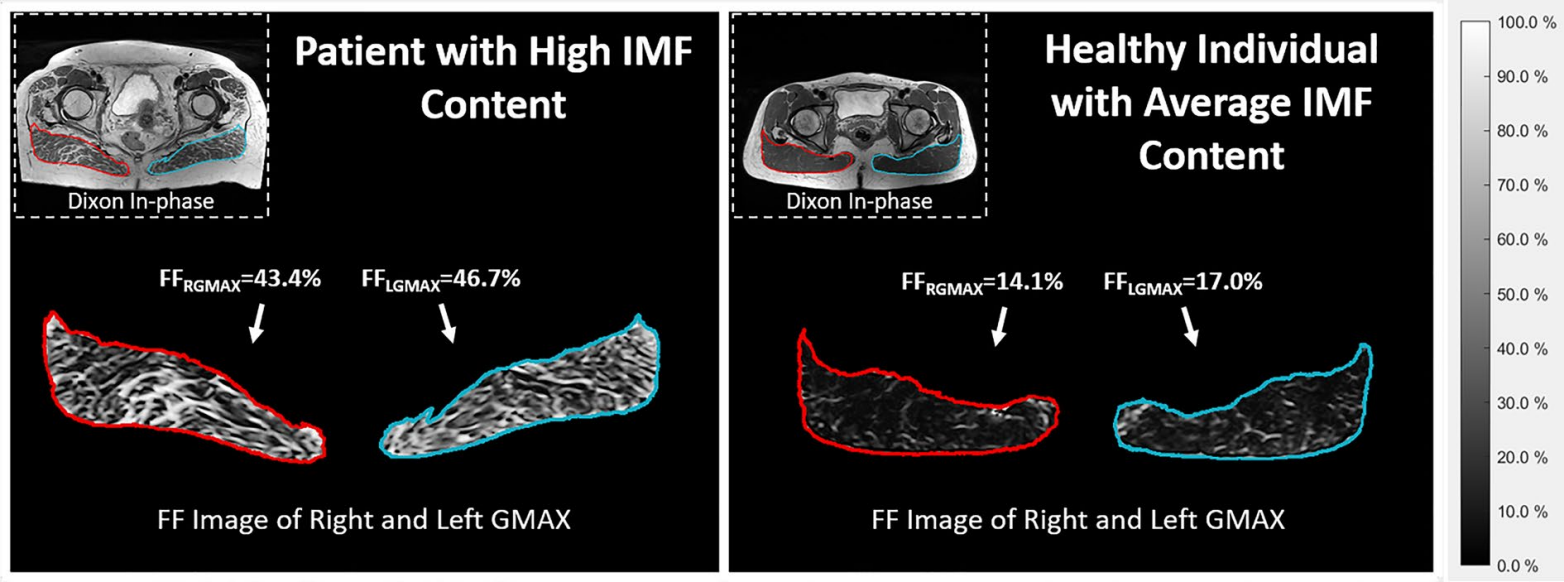
Example cases for different levels of FF. Axial slice of the FF image of right and gluteus maximus for a subject with high fat infiltration from the patient group and for a healthy subject of the medium activity group with a GMAX FF close to the average for this group. The FF values of each muscle are included in the images. On the top left corner of each image, the in-phase Dixon MR image of each case is shown.

For the HS, two outliers with a GMAX FF higher than 26.1% (Q3 + 1.5*IQR of all the healthy subjects) were found, while 10 of the 19 subjects in the patients group exceed that level of FF. In Fig. 3, we show axial slices of a subject with high IMF content from the patients group and of a subject with average IMF infiltration from the medium activity group, where a large difference in the FF signal between the two images can be seen.

### Differences between right and left gluteus maximus

**Figure 4:**
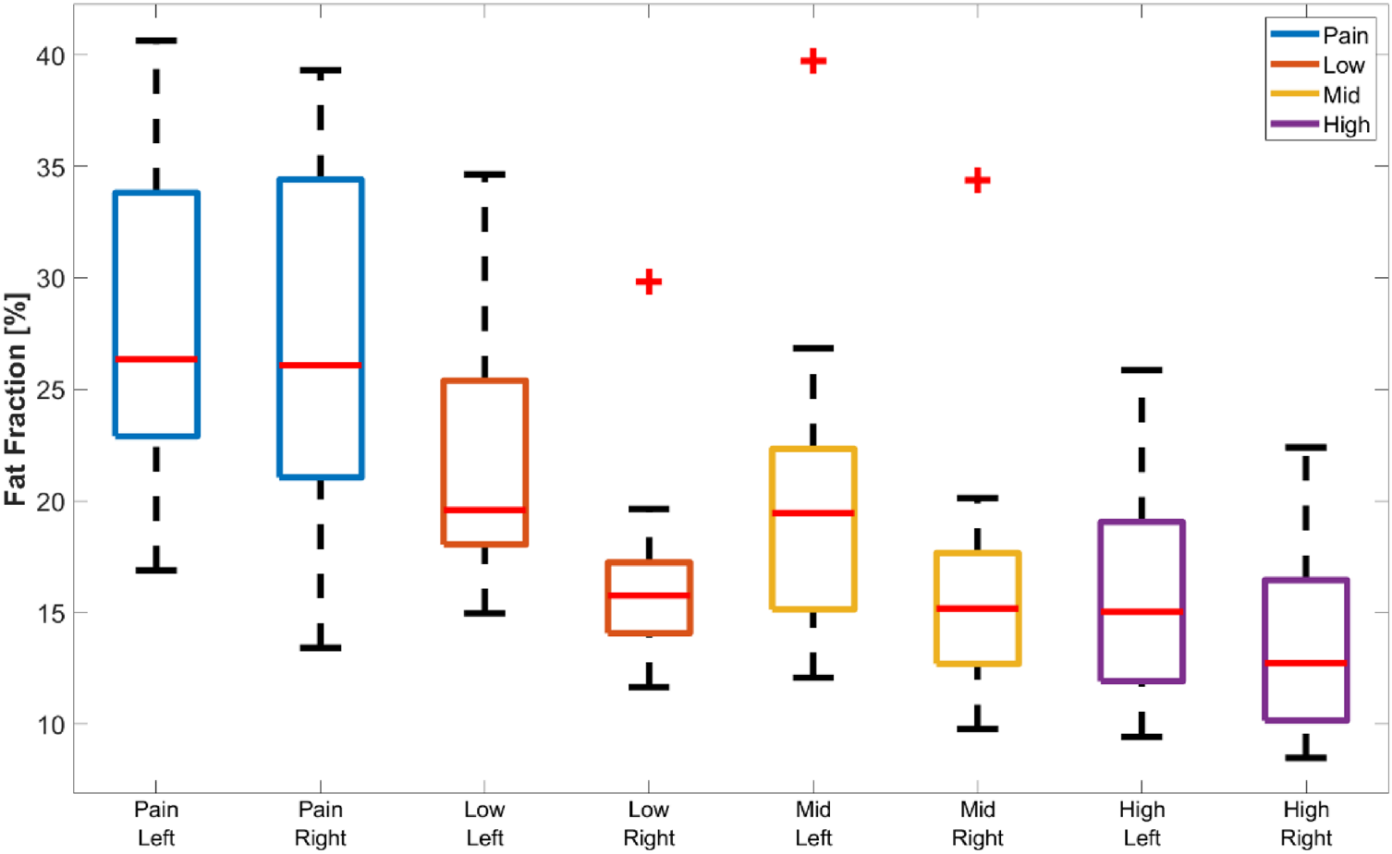
Boxplots of GMAX FF values for left and right side for each group under study. On each box, the central mark is the median, the edges of the box are the 25th and 75th percentiles. The outliers are plotted individually. Left FF was significantly higher than right in the low and mid activity groups (*p* < 0.05).

In the healthy subjects group, the GMAX FF was 3.3% higher in the left than in the right (median, Wilcoxon signed-rank test *p* < 0.001). When looking at the groups separately, left FF was significantly higher in the low (*p* = 0.006) and mid (*p* = 0.01) activity groups, but not for the high activity (*p* = 0.09). Figure 4 shows boxplots with FF per side for each group where these differences are visible. For the patients group, the FF values were not different between left and right. In addition, we did not find any significant difference when comparing the side with the affected hip and the asymptomatic side (median − 0.5%, Wilcoxon signed-rank test, *p* = 0.90).

### Hierarchical linear regression analysis of predictors of fat fraction

We studied all the potential covariates for FF using a hierarchical linear regression analysis of FF. We included as predictor variables gender, BMI, age, level of physical activity, the OHS grading, the muscle side and the pain side. The results of the hierarchical linear regression analysis are reported in Table 3, where the final model with all the variables that increased the R^2^ were included. Female gender, high BMI, low levels of physical activity and left side were predictors of increased FF. The OHS grading and the pain side were not predictors of FF.

**Table 3:** Results of a hierarchical linear regression analysis of predictors of fat fraction.

Predictor variables	Model*	R^2†^
Gender (male)	− 3.8 (*p* < 0.001)	0.118 (0.118)
BMI	0.9 (*p* < 0.001)	0.331 (0.449)
Age	0.1 (*p* = 0.02)	0.080 (0.529)
Activity level	Pain = 7.2 (*p* < 0.001)	0.085 (0.614)
	Low = 5.2 (*p* < 0.001)	
	Mid = 1.8 (*p* = 0.13)	
Side (right)	− 3.0 (*p* < 0.001)	0.037 (0.651)

The coefficients with their respective p-values are reported for each predictor variable for each model. The coefficients are in FF percentage units.

*(FF ~ 1 + gender + BMI + age + activity + side). OHS and pain side were also tested but were not predictors of FF.

^†^The accumulated R^2^ after the inclusion of each variable are reported between parentheses.

For the levels of activity variable, hip pain and low physical activity were statistically significant predictors of fat infiltration. This analysis shows that the level of physical activity impacts on the IMF content of GMAX, after including gender, BMI and age as covariates.

The post hoc statistical power analysis indicated that the power of physical activity as a predictor of GMAX FF was 0.85 for a significance level of 0.05.

### Gluteus maximus volume and physical activity

**Figure 5:**
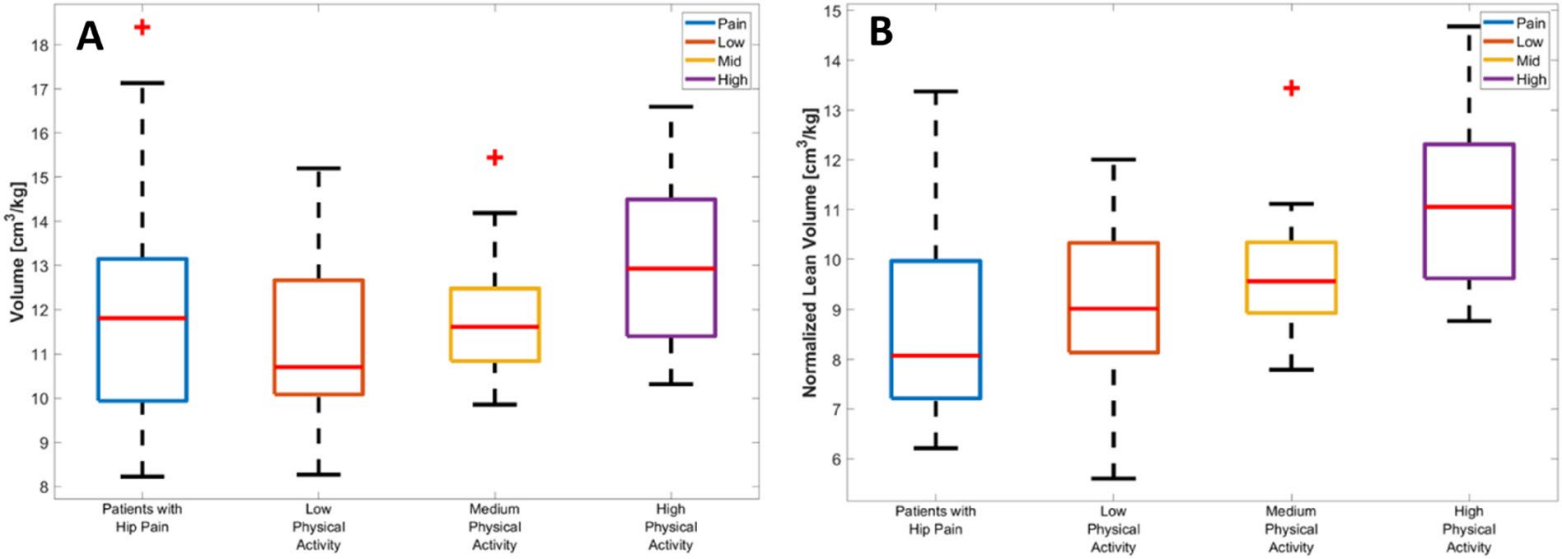
Volume metrics for each activity level group. Boxplots of (**A**) normalized volume and (**B**) lean normalized volume values for each group under study. On each box, the central mark is the median, the edges of the box are the 25th and 75th percentiles. The outliers are plotted individually. In A), the high activity group was significantly higher than the low activity and pain groups. In (**B**), the high activity group was significantly higher than the other three groups (*p* < 0.05).

In order to complete the analysis, we have also included results for volume. In Fig. 5, we show boxplots of normalized volume (A) and normalized lean volume (B) for each of the groups under study. The normalized volume of GMAX was larger for the high activity group than for the patient (*p* = 0.03) and low activity groups (*p* = 0.004), but not for the mid group (*p* = 0.06). The normalized lean volume was larger in the high activity group compared to all the other groups (*p* < 0.001, *p* = 0.002 and *p* = 0.02). There was not any statically significant differences between the patient, low and medium activity groups.

### Gluteus maximus intramuscular fat and volume as predictors of physical activity

We tested GMAX FF and volume, together with the other covariates, as predictors of physical activity using a hierarchical logistic regression (Table 4). The variables associated with being physically active were age (OR = 1.04, *p* = 0.03) and GMAX FF (OR = 1.22, *p* < 0.001). This means that the odds of being physically inactive are 1.22 times higher among subjects with higher GMAX intramuscular fat. GMAX volume was not associated with the probability of being physically inactive.

**Table 4:** Results of a hierarchical logistic regression analysis of variables associated with being physically inactive and with hip pain.

Model	Predictor variables	Odds ratio	95% CI	*p* value
No active versus active*	Gender	2.06	0.83–5.14	0.12
	BMI	0.93	0.81–1.06	0.28
	Age	1.04	1.00–1.08	0.03
	GMAX FF	1.22	1.11–1.33	< 0.001
	GMAX volume	0.8	0.64–1.00	0.06
Hip pain versus healthy^†^	Gender	0.71	0.12–4.01	0.52
	BMI	1.21	0.92–1.59	0.02
	Age	1.12	1.04–1.20	< 0.001
	GMAX FF	1.12	0.95–1.33	0.05
	GMAX volume	0.78	0.48–1.27	0.11

The first model estimates the odds of being physically not active or active for each variable, while the second the odds of having hip pain or a healthy hip. The odds ratios, 95% confidence intervals and *p* values are reported.

*(Pain + low activity) versus (medium + high activity).

^†^(Pain) versus (low + medium + high activity).

Finally, we also tested GMAX FF and volume as predictors of hip pain. In this case, BMI (OR = 1.21, *p* = 0.02) and age (OR = 1.12, *p* < 0.001) were associated with hip pain, while GMAX FF was only borderline associated with it (OR = 1.12, *p* = 0.05).

## Discussion

This is the first study to quantitatively measure IMF content of GMAX in subjects with different levels of physical activity. We found that hip pain and low physical activity were predictors of fat infiltration in GMAX, along with gender and BMI. The high activity group had considerable lower GMAX IMF content than the other HS groups, but this difference was only statistically significant between the high and low activity groups. The IMF content in patients with very low physical activity due to hip pain was significantly higher than in healthy subjects. These are clinically relevant findings since they suggest that IMF infiltration in GMAX could be used as a marker for mobility and muscle health, which can be measured automatically by estimating GMAX FF from Dixon MRI of the pelvis.

The main limitation of this work is a relatively small number of subjects employed in this study given all the covariates for IMF, such as gender and BMI. In addition, the number of male and female individuals were not even in every activity group. Nevertheless, we found statistically significant differences between the activity groups and the hierarchical linear regression analysis showed that all these variables, including the level of activity, were predictors of IMF.

### Measurement of intramuscular fat in gluteus maximus

The use of 3D labels and Dixon MRI allowed us to overcome some of the limitations of other IMF measurement techniques. 3D labels can measure IMF in the full or a whole section of the muscle instead of using CSAs from only a few slices^2,16,22^, which then needs extrapolation or assumes uniformity. Furthermore, when combined with Dixon MRI, a quantitative measurement of IMF can be obtained with very low cross-talk with IMAT, in contrast to intensity-based segmentation of adipose tissue from other sequences, such as T1-weighted, where usually IMF and IMAT are not treated separately^18,30,43^.

### Gluteus maximus intramuscular fat and physical activity

We have shown that a decreased level of physical activity is linked to an increase of IMF content in GMAX, even for healthy subjects. These results agree with a previous study looking at fat infiltration in the thigh and calves muscles of healthy subjects, where higher fat infiltration was found in a group that went through 4 weeks of unilateral limb suspension compared to a control group^16^.

There was a marked difference in GMAX FF values between patients with hip pain and healthy subjects, but we also found significant differences for different level of physical activity. The low activity group consisted of young and slim subjects with a low mean BMI of 22 kg/m^2^, therefore BMI did not affect the comparison between healthy subjects. A limitation of our study is that the group of high physical activity was formed mainly by men (M = 16, F = 4), potentially reducing the mean FF for this group as female gender was a predictor of higher FF. Another potential limitation is that the hours of physical activity were obtained by a self-reported questionnaire. Despite these limitations, the differences between the levels of physical activity were statistically significant when taking into account all the covariates and the tests performed had good statistical power.

Another factor to take into consideration is that endurance athletes have a higher content of intramyocellular lipids (IMCL) than other athletes and sedentary individuals as this is an adaptation in response to endurance training^44^; while loss of muscle strength, muscle atrophy and a sedentary lifestyle is associated with increased extramyocellular lipids (EMCL)^45,46^. The Dixon FF signal has contributions from both IMCL and EMCL^47,48^, therefore a potential lower content of EMCL in the high activity group, with several multiple marathon runners in it, could be harder to distinguish due to increased IMCL deposits.

### Differences between right and left gluteus maximus

The GMAX FF was higher in the left side than in the right for the healthy subjects, very likely due to leg dominance. We did not record footedness in our study, but the vast majority of the population is right-footed^49^. On contrary, we did not see any difference between sides on the patients group, probably due to the effect of the hip pain being dominant over footedness. When comparing the pain side with the asymptomatic side, the FF differences were not significant. However, only eleven out of 19 had unilateral hip pain, making this comparison statistically challenging.

### Effects of gender, age and bmi on gluteus maximus intramuscular fat

Female gender was a predictor of higher GMAX IMF. These gender differences have also been observed in studies using needle-biopsies of quadriceps femoris^50^ and Dixon MRI of the thigh^51^. Age was not a strong predictor of IMF content of GMAX in our study sample, where all the subjects were younger than 63 years and the mean age was 50 and 33 years for the patient and HS groups respectively. This agrees with other studies were an increase of IMF with age was mainly observed in individuals aged 70 years or more^51,52^. On the other hand, BMI was a moderate predictor for GMAX IMF and this has been also previously observed in the thigh muscles^51^.

### Baseline values for gluteus maximus intramuscular fat in healthy subjects

The reported FF values for healthy subjects can be used as baseline values for GMAX IMF content. Normative FF values have been previously reported for gluteus medius and minimus^52^, but not for GMAX. Mean FF values of 8–9% were found for these two muscles, and no significant differences were found between genders^52^. On the other hand, we obtained a mean FF value of 14.8% for male and 20.4% for female healthy subjects. This would indicate that FF values are higher for GMAX than for the other gluteal muscles. These differences, which are usually noticeable by visual inspection of MR images, can be related to the different function and fibre composition of the gluteal muscles. Differences in IMF content within a muscle group have also been detected in other studies^3,22,30^.

### Gluteus maximus intramuscular fat in patients

The GMAX FF was significantly higher in the patients than in the healthy subjects, which can be attributed to a very low physical activity due to hip pain. Only one of the 19 patients’ scans were reported with fatty atrophy, however a considerable high GMAX IMF content (defined as a FF higher than the outlier level of the HS) was measured in 9 patients. This would indicate that GMAX FF from MRI can detect and quantify fat infiltration in early stages of hip disease or injury.

GMAX FF can be potentially used to study and assess quantitatively the progress of mobility and muscle health in patients with certain pathologies such as osteoarthritis (OA) as well as the effects of rehabilitation practice. In this regard, a more pronounced fatty infiltration in the gluteal muscles was related to the severity of OA, when fatty infiltration was limited to a qualitative assessment^25^. In addition, GMAX fat infiltration was linked to falls in the elderly using computed tomography (CT)^27^ and Goutallier’s staging^19^ to quantify IMF. Our results and these other applications show that automated assessment of GMAX IMF can be a clinically relevant marker.

### Gluteus maximus volume and physical activity

In this work, we focus on IMF, but we have also included results for volume, as this is another marker of interest when assessing muscle health. The normalized volume of GMAX was statistically significantly larger for the high activity group than for the patient and low activity groups. However, the differences were much smaller than for FF as it can be seen in Figs. 2 and 4. As expected, when using the lean volume as a metric for muscle size, these differences were proportionally larger. Different to FF, GMAX volume was not a predictor of subjects physically active. Based on these results, FF seems to be a more robust muscle health marker to predict muscle degradation due to low physical activity.

## Conclusion

In this work, we used an automated tool to compute FF in GMAX muscle as a measure of GMAX IMF content in four groups of subjects with different levels of physical activity. We report novel quantitative data of GMAX IMF content for these groups that ranged from patients with reduced physical activity due to hip pain to highly active subjects reporting more than 8 h of physical activity per week, included experienced marathon runners. The group of patients with hip pain had a significantly higher IMF content than the three groups of HS and the group of high levels of activity had a significantly lower IMF content than the low activity group. We found that hip pain, low physical activity, female gender, BMI and left side are predictors of GMAX fat infiltration, even within healthy subjects. These results show that automated measurements of GMAX FF could be used to quantitatively assess muscle health, mobility and sarcopenia in clinical trials and clinical practice.

## Supplementary Information

Supplementary Information.
