# Supplementary material for: Intramuscular fat in gluteus maximus for different levels of physical activity
Source: Sci Rep. 2021 Nov 1;11:21401. doi: 10.1038/s41598-021-00790-w (PMC8560940; doi:10.1038/s41598-021-00790-w)
Supplement: Supplementary file 1 — Supplementary Information. [file 41598_2021_790_MOESM1_ESM.docx]

**Intramuscular Fat in Gluteus Maximus for Different Levels of Physical Activity**

Martin A. Belzunce, Johann Henckel, Anna Di Laura, and Alister Hart

**Supplementary Table S1 – Patient group details**

Table S1. Side of the hip pain and MRI primary finding for each subject in the patients group.

| Patient | Side of Hip Pain | OHS^†^ | Primary Finding in MRI |  |
| --- | --- | --- | --- | --- |
| 1 | Right | Mild | Gluteus medius insertional tendinopathy. |  |
| 2 | Right | Satisfactory | Subchondral cyst in right acetabulum. |  |
| 3 | Bilateral | Mild | Gluteus medius insertional tendinopathy. |  |
| 4 | Bilateral | Satisfactory | L4-L5 intervertebral disc pathology (referred pain). |  |
| 5 | Right | Mild | Gluteus medius insertional tendinopathy. |  |
| 6 | Left | Moderate | Left: gluteus medius insertional tendinopathy. Right: echondroma in femoral head. |  |
| 7 | Right | Severe | Right hip OA (labral tear). |  |
| 8 | Right | Severe | Significant degenerative hip with labral cyst. Right: fatty atrophy in gluteal muscles. |  |
| 9 | Right | Satisfactory | Mild right hamstring tendinopathy. |  |
| 10 | Bilateral | Mild | Slight bilateral trochanteric bursitis. |  |
| 11 | Bilateral | Moderate | Bilateral gluteus medius insertional tendinopathy. |  |
| 12 | Right | Satisfactory | Mild left hamstring tendinopathy. |  |
| 13 | Bilateral | Satisfactory | Bilateral sacroiliitis. |  |
| 14 | Right | Moderate | Right hip OA. |  |
| 15 | Right | Moderate | Right hip OA. |  |
| 16 | Right | Mild | Right femoroacetabular impingement. |  |
| 17 | Bilateral | Moderate | Bilateral hip OA. |  |
| 18 | Bilateral | Mild | Bilateral hip OA. |  |
| 19 | Left | Mild | Left labral tear. |  |
| ^†^ Oxford Hip Scores grading: Severe (0-19), Moderate (20-29), Mild (30-39), Satisfactory (40-48). | | | | |
